# Mixed experiences with online learning among neurodiverse youth: Evidence from video recordings of a longitudinal study

**DOI:** 10.1371/journal.pmen.0000167

**Published:** 2024-10-24

**Authors:** Celeste Campos-Castillo, Elise Atkinson

**Affiliations:** Department of Media and Information, Michigan State University, East Lansing, Michigan, United States of America; Assiut University, EGYPT

## Abstract

Among those for whom there were heightened concerns regarding the pivot to online learning during the COVID-19 pandemic were neurodiverse youth. Despite the preponderance of research into online learning during the pandemic, relatively few studies have directly observed the experiences of neurodiverse youth. We present findings from a longitudinal study in which we qualitatively analyzed using an idiographic approach the nearly 2,000 minutes of video recordings of 9 neurodiverse youth learning digital art design via the Zoom platform. The themes we developed from the patterns observed suggest how online learning may both enable and inhibit neurodiverse students’ syncing their communication with ongoing conversations with others, achieving intersubjectivity (shared understanding) with others, coping with frustration, and personal disclosures for building rapport with others. Notably, we observed evidence suggestive of benefits and detriments of online learning for each neurodiverse youth. We discuss implications for the design and implementation of online learning opportunities for neurodiverse youth.

## Introduction

The widespread disruption when U.S. schools pivoted to online learning in 2020 to curb the spread of SARS-CoV-2 raised concerns about potentially harmful effects on youth [1, 2]. Among those for whom there were heightened concerns were neurodiverse youth [3–5], which we refer to as individuals who have neurological and cognitive functions not considered typical, including autism spectrum disorder (ASD), attention-deficit/hyperactivity disorder (ADHD), and dyslexia. While distinct from one another, neurodiverse groups share some similarities in experiences, such as facing stigma within learning settings because they neither communicate nor perform speech and motor tasks in ways considered typical [6–9]. Concerns about pivoting to online learning for neurodiverse groups included diminished social connectedness, disruptions to routines, losing access to services, and, despite longstanding assumptions that some neurodiverse groups (the ASD group) are “naturals” at using technology [10], the inability to navigate online platforms successfully [3–5, 11]. While several studies show evidence confirming that these concerns materialized [12, 13], others show contradictory evidence, with some depicting within the same set of findings a complex set of outcomes that were both positive and negative for neurodiverse youth [4, 5, 11, 14].

The mixed findings may be due in part to the diverse approaches to online learning, with several approaches being investigated for their effectiveness in supporting neurodiverse youth even before the COVID-19 pandemic. The different approaches entailed differences in modalities and topic domains. Modalities included both virtual reality initiatives and video calls across various platforms, while topic domains included group challenges, physical exercise classes, mentoring sessions, among others [15–18]. Online learning experiences resulted in a range of positive outcomes, such as improvements in life skills, adaptation to remote learning settings, and increased levels of interpersonal communication and physical exercise, although improvements varied between individuals [15–17]. Concerns were raised too, with challenges related to access and engagement with online learning platforms reported, particularly during the COVID-19 pandemic [19–21]. These issues underscore the complexity of addressing the diverse needs of neurodiverse individuals through online learning and highlight the importance of ongoing research into their design and implementation.

Another likely contributor to mixed findings are the diverse sources of data. Completion of self-reports by neurodiverse youth presents several challenges, including the lack of valid, reliable, and accessible scales tailored for the population [22–26]. To overcome this obstacle, researchers may turn to guardian-reported outcomes, but these may be biased. For example, guardian-reported self-esteem tends to be lower than youth-reported among neurodiverse youth, but not for neurotypical youth [27]. Less typically collected was observational video data included in qualitative analyses [15, 18, 28, 29], which is what we obtained in the current study.

We advance scholarship on experiences with online learning among neurodiverse youth by reporting on findings from a longitudinal study in which we qualitatively analyzed an underutilized data source: video recordings of neurodiverse youth engaged in learning. Specifically, the neurodiverse students sampled were enrolled in a five-week workshop to learn digital art design. We leverage the study design using an idiosyncratic approach [30, 31] by showing positive and negative experiences with online learning *within each neurodiverse student*. Thus, findings indicate online learning is not a monolithic experience, but rather changes based on the situations that individuals encounter. Moreover, by relying on video recordings, we overcome limitations of relying on self-, caregiver-, and teacher-reports. We discuss the implications for designing and implementing online learning opportunities that diminish the likelihood of negative experiences while enhancing the likelihood of positive experiences for neurodiverse youth. Given the aforementioned interest among scholars and practitioners in online learning for neurodiverse youth that predated the pandemic, such insights are likely to remain relevant now that the public health emergency has ended.

## Methods

### Ethics statement

This study received approval as an expedited study from the Institutional Review Board at the University of Wisconsin-Milwaukee (protocol: 22.304). Formal written consent and assent were obtained.

### Study context

The non-profit organization that hosts the digital art design workshops from which we obtained data, Creative Oasis (a pseudonym), is located in a mid-sized city in the Midwestern region of the United States. The workshops are among several digital art-focused programs targeted specifically for neurodiverse individuals. Data were collected November 2022 to May 2023, a time in which Creative Oasis was offering both in-person and online workshops.

To be eligible to enroll in a workshop, an individual requires either a professional diagnosis or the professional judgment of Creative Oasis staff that they have characteristics consistent with neurodiversity. The latter enables addressing biases in access to clinical professionals who formally diagnose and in the diagnostic tools that they use [32, 33]. Of those who meet this criteria, Creative Oasis staff further distinguish between those with and without the capability to work with a mentor who shows them how to design digital art during the workshop. Specifically, a neurodiverse individual is eligible to enroll if an interview with Creative Oasis staff suggests their social communication and any presence of repeated, restricted behaviors are consistent with a Level 1 (requiring support) or 2 (requiring substantial support) diagnosis for autism spectrum disorder [34]. While students presented with characteristics consistent with neurodiverse conditions besides autism spectrum disorder, Creative Oasis staff used this criteria as a guideline to determine who was likely able to sit for the entirety of a workshop session and communicate successfully with their assigned mentor about how to use the software to complete their art design project. The use of the criteria is consistent with research suggesting distinct neurodiverse groups communicate and perform speech and motor tasks in ways that are not considered typical [6–9].

### Neurodiversity community outreach

Prior to obtaining approval for study procedures, the researchers conducted outreach activities with neurodiverse youth and their families to obtain input on the study design and gain their trust. The lead author attended in-person and online art design workshops held by Creative Oasis to introduce herself and discuss the proposed study. She held a focus group that met for three one-hour sessions with three guardians of neurodiverse students attending a workshop to obtain their iterative feedback on the wording of consent/assent forms, outcomes to measure, and best practices for recording workshop sessions. To maximize input from families who were unable to sit for an in-person focus group session, she scheduled 30 to 60-minute telephone and Zoom conversations with interested guardians and also conducted brief (5–10 minutes) informal conversations with families and students before, during, and after workshop sessions.

An important outcome of the community outreach activities was text in the consent/assent forms to emphasize to neurodiverse youth in accessible language that their participation was completely voluntary and that they may withdraw after enrolling. While such text is a requirement in consent/assent forms, guardians expressed concerns that neurodiverse youth who enroll in the study may feel obligated to enroll and continue, despite feeling overwhelmed by study procedures (like the recordings). Accordingly, both the consent (for adults) and assent (for minors) forms for neurodiverse youth stated at the beginning: “We’re inviting you to be a part of a research study. A research study is a way to learn new things. You don’t have to be in this study. It is up to you. If you say yes now, but change your mind later, that’s ok too. Just let me know.”

### Workshop structure

During a Creative Oasis workshop, the neurodiverse student and their assigned mentor work collaboratively to design digital artwork, which would then be printed on poster board for the student to take home. The mentor is an adult who demonstrates proficiency with the design software that the student uses in the workshop, described momentarily. The student selects the subject of the digital artwork and may use the five sessions to work toward creating either a single artwork or multiple artworks and then decide which one to print. A typical workshop comprises five 75-minute sessions, with each session meeting weekly at the same time and day. To accommodate school schedules, sessions are held either on weekday evenings or during the day on weekends. While Creative Oasis offers workshops in other configurations (e.g., five 75-minute sessions occurring throughout one week over the summer), we only observed students enrolled in the typical configuration to develop general principles.

The student decided on the design software, which was either for creating 2D or 3D art. The latter was always Blender and the former was Adobe Illustrator (hereafter, Illustrator), Adobe Photoshop (hereafter, Photoshop), or Procreate. Blender, Illustrator, and Photoshop were run on either a desktop or laptop computer, while Procreate was run on an Apple iPad (hereafter, iPad). When a student in a 2D art workshop elected to use Procreate, the student and mentor would each log into the Zoom session using both an iPad and a webcam-enabled computer, thereby allowing each the option to screenshare the iPad they were using while simultaneously sharing their face via the computer. Most often the student would screenshare their iPad while both the student and mentor shared their face via their computers; the mentor would only screenshare their iPad as needed.

Each of the five sessions begins with a prep meeting in the main Zoom room. The prep meeting begins approximately 15 minutes prior to a session’s official start time with the Creative Oasis staff who are tasked with providing support for the session. Staff present include the workshop moderator, software support technicians, mentors assigned to each student, and backup mentors who would step in when a student’s usual mentor had to cancel or exit a session unexpectedly. The researcher(s) who would record sessions, which included the two authors and research assistants, would also log in at this time and introduce themselves to the group. The workshop moderator granted the researcher(s) co-host privileges, enabling them to record and enter or exist breakout rooms as needed. Students would log in to the Zoom session and wait in the waiting room for the session to start.

A session starts with the moderator welcoming everyone and providing them with an icebreaker question, to which the students could volunteer a response by unmuting their microphone or typing into the chat. The moderator would then send each student and their mentor to a Zoom breakout room, where the two would work together on the student’s artwork. The researcher would join the breakout room, turn off their video to minimize disruption, and record. The time spent working on the student’s artwork in the breakout room ranged from 45 to 60 minutes. Throughout this time, the moderator would switch between breakout rooms to monitor progress and intervene as necessary, such as assisting with technical problems that arose.

For the final 10 to 15 minutes of each session, everyone returned to the main Zoom room so that each student could screenshare and show the progress they made on their artwork. While each student presented, the moderator would probe about the process of creating the artwork, such as by asking whether the student had to solve any problems or learned new software tools. Each student’s presentation lasted approximately 2 to 3 minutes and ended with other students and mentors offering praise and suggestions for next steps.

### Participant recruitment

Creative Oasis shared with us the names, age, and contact information of neurodiverse students who registered for their art design workshops. For minors, they also shared the names and contact information of the student’s guardian. Prior to a researcher making contact, Creative Oasis staff sent students and guardians an email to let them know that they would receive an invitation to participate in a research study and that their participation was completely voluntary.

Beginning one week prior to the first session of a workshop up to its start time, researchers emailed eligible students and their guardians up to three times to invite them to participate in the study. The email provided them with an overview of the study and links to consent/assent forms. Parents/guardians and students over the age of 18 provided written consent and minors provided written assent.

### Sample

Video recordings were obtained from observing 9 neurodiverse students enrolled in a typical 5-session art design workshop. The students ranged in age from 10 to 25, with 8 males and 1 female. Each student was recorded at least once. Instances in which a student was not recorded included technical malfunction, student absence, and lack of consent (from adult students and from guardians for students who were minors). In total, there are 30 session recordings, representing approximately 2,000 minutes of video recordings.

Sessions were transcribed automatically using both Zoom and Microsoft. A research assistant reviewed the video recordings and transcripts for accuracy. The assistant selected pseudonyms to use in place of real names and redacted any personally identifiable information from the transcripts.

### Analysis

Analysis began by the authors familiarizing themselves with the data and discussing potential differences in student experiences based on the art design software used. The discussion was further facilitated by the fact that both authors were involved in recording sessions, and thereby were familiar with a breadth of experiences. The backgrounds of the authors are also complementary. Specifically, the first author is an expert in qualitative analysis of data from youth while the second is an expert in art design software. Both are familiar with neurodiversity through community outreach.

Both authors began analyzing the transcripts using thematic analysis [35]. The thematic analysis began with open coding, whereby each author independently reviewed the transcripts of all recorded sessions for a neurodiverse student and developed codes to summarize patterns. This gave way to an idiographic focus [30], in which the holistic experience of an individual participant is the focus of the analysis. The authors met weekly to review the codes they developed and discuss axial codes, which summarize themes in the codes. During these meetings, they continued to discuss potential differences in experiences based on the art design software used.

The authors achieved thematic saturation after reviewing the ninth transcript, in that no new codes were developed [36]. This threshold point was confirmed further during weekly meetings to discuss the remaining transcripts. The remaining transcripts were split evenly between the authors so that only one author analyzed and applied the codebook to a transcript. The authors continued to meet weekly to discuss their analysis of a transcript, in which it was revealed that no new codes were needed to summarize patterns.

For each theme, we reviewed representative excerpts from each individual transcript to consider the data as a whole. We used the constant comparative method [37] to refine the contours of each theme. The resulting four themes we developed represent affordances of online learning, with each theme depicting both positive and negative outcomes for the neurodiverse research participants. Given the depth of detail gleaned from recording participants longitudinally, each participant had at least one excerpt that typified each theme, though it was not always the case that each participant had excerpts across the full breadth (positive and negative outcomes) of a theme. To preserve the idiographic focus and leverage the complexity and texture of the details, we selected for presentation of each theme one participant whose experiences typified the full breadth of a theme. This enables depicting instances *within each student* in which the online modality likely enriched their experiences and other instances *within the same student* in which it was likely a detriment. Thus, an advantage of an idiographic approach is demonstrating how online learning is not a monolithic experience, but rather can change dynamically for any given student because the situations in which they use the technology changes [31]. We present vignettes for four students that summarize how online learning was both a benefit and detriment to each.

## Results

### Syncing communication

Gerardo is a male in his late teens who relies on the Rapid Prompting Method to communicate [38], with his mother as his aide. His mother holds a letter board in the air and asks him a question, to which he replies by pointing to the letters to spell out his response. As Gerardo points to a letter, his mother says the letter out loud and then states the word that was spelled. Occasionally, she asks Gerardo to say a word out loud on his own and he succeeds. For his workshop, Gerardo is working with his mentor to design artwork using Procreate.

Despite video conferencing platforms like Zoom providing the affordance of synchronous communication, Gerardo’s use of the Rapid Prompting Method introduces disruptions into the flow of communication with his mentor. For example, Gerardo’s first session begins with his mentor asking his thoughts on school and education. The mentor gets no immediate response, so then asks, “So have we given any thought to what our project will be?” Gerardo is pointing to the letters on the board that his mother is holding up in the air. Eventually, his mother states, “OK, so an answer to your last question, Gerardo just said education is great.” Gerardo’s use of synchronous channels of communication, like video conferencing platforms, are experienced asynchronously because his reliance on communication aids like the Rapid Prompting Method produces responses out of sync with the flow of others’ conversation. Accordingly, any benefits of synchronous over asynchronous communication for student outcomes [39] risk getting diminished.

At the same time, however, Gerardo has Zoom experiences that are typical of others, in which his reliance on a communication aid like the Rapid Prompting Method appears less disruptive to the flow of conversation. With the rapid shift toward videoconferencing to maintain physical distance during the COVID-19 pandemic, unintentional “Zoom bombing” by young children, roommates, and pets became frequent to the point that people became less likely to feel embarrassed when it happened [40]. A “Zoom bombing” incident occurred while Gerardo and his mentor were discussing what he would add to his artwork, which was comprised of a series of musical instruments:

Mentor: Do you have an instrument in mind?

Gerardo’s mother (while Gerardo points to letters on the board): Go ahead.’V’. Can you say the letters? ‘V,’ ‘I,’ ‘O’. Tell [mentor] what does that spell.

Gerardo: Violin

Mentor: Violin ok awesome. Well, if I remember right, we did pick a picture of a violin for your name tag. Do you want to use that picture? We could also find another one. Your call.

Gerardo’s mother (while Gerardo points to letters on the board): ‘A,’ then ‘N’. Ohh there’s her kitty.

The mentor’s cat had unexpectedly jumped onto her desk and was visible in the Zoom meeting. Gerardo continued pointing to letters on the board while the dialogue continued:

Mentor: Yes, she’s very stinky right now. But here she is.

Gerardo’s mother: Oh, look at how pretty she is.

Mentor: I know she’s gorgeous and she’s such a monster.

Gerardo’s mother (to Gerardo): Did you see her? Isn’t she beautiful?

Mentor: I just came home for the first time today, so she’ll be asking for my attention this entire time.

Gerardo’s Mother: That’s good, good. We’ll have to see her again. He said another violin another, he said.

Like other instances of unintentional “Zoom bombing” from household members [40], the subject of conversation turned to the “bomber,” in this case the mentor’s cat. The break in the conversation afforded Gerardo the time to catch up and continue spelling his response to the mentor’s question, which his mother relayed was that he wanted to pick another picture of a violin. Because of the interruption from the cat, which is unlikely in a more formal in-person setting, Gerardo was able to work toward syncing his response with the conversation about what to do next for his artwork.

### Coping with technology

Kaya is a White male in his early twenties who chose to work with Blender during his workshop. When faced with an unexpected technology problem, Kaya appears visibly frustrated. However, while other neurodiverse youth may labor with regulating frustration [41, 42], he appears to manage it well because he quickly shifts into making verbal statements that declare he can fix it.

The online learning format seemed to enhance Kaya’s ability to cope with frustration over technology problems. At the start of the third session, Kaya’s screenshare shows a three-dimensional alien he has been designing. The mentor says, “So I remember last time we talked about possibly wanting to make this glow. Do you still want to do that?” Kaya responds affirmatively. The mentor then uses the annotation tool to draw a small circle around the rendering option to the right to direct Kaya to click on and expand it to see whether the option they are looking for, the bloom option, appears. Kaya follows through with the direction, but the bloom option does not appear. This is due to Blender being in the default render setting, ‘Cycles’, which does not offer the bloom option. The mentor uses the annotation tool to again direct Kaya to click on and expand another option, and Kaya obliges, but still no success in finding the bloom option. For approximately the next four minutes, the process of the mentor directing Kaya to click around and Kaya obliging repeats as the mentor tries to recall how to make the alien glow.

Kaya begins to rub his hand on his face and appears frustrated. The mentor tells Kaya, “All right, one moment and I will be right back with you.” One minute of silence between them passes while the mentor multitasks by looking up online on her own computer how to access the bloom option. The mentor finds the information online and successfully directs Kaya toward making his alien glow. Thus, while multitasking during online meetings may at times pose distractions and inhibit engagement with others, it can also improve wellbeing by boosting productivity, especially when the multitasking is to look up information that would enhance the meeting experience [43]. In Kaya’s case, the mentor’s multitasking with her own computer appears to have curtailed his growing frustration with being unable to locate the tool to make his alien glow.

Later in the same session, technology not only obstructs Kaya’s ability to cope but further aggravates the situation. Kaya has now designed two aliens and is trying to move one by selecting all the meshes and moving them on one axis. He struggles to do so and exclaims while throwing his hands in the air, “I don’t know what to do.” As is typical of Kaya’s character during the workshop, he quickly reassures himself and says, “I do know what to do,” and his mentor follows suit by saying, “You got this.” He tries again, unsuccessfully, and states he does not know what to do. The mentor then intervenes and states, “I might have a solution for us. I’m going to request remote control access and I’m going to see if I can do it.” The mentor gains remote control access, but as she moves the alien forward, she unintentionally made it oversized. Kaya suggests using his keyboard to undo what the mentor did and try again on his own to select the meshes using keyboard shortcuts, but continues to struggle: “And no, no. Whenever I press G this happens.” At the moment, a viewer cannot see what Kaya is referencing. Eventually, we learn that the key combination he was pressing on the keyboard initiated the Game Bar, but because Kaya had only selected to screenshare the Blender app, a viewer could not apprehend the problem fully. Kaya continues to try to solve the problem on his own, but then pleas for his mentor to help:

And but but but. We can do this. Oh, and then ‘G.’ What is it? I should be quiet. No, what’s happening? No, I can do this, I swear. Wait a minute. I’m pressing the wrong thing. No, something’s wrong. I need help. What do you want me to do? No, I can stop this… No, no, no, this is bad. I can’t hear you. I can’t understand you. You’re glitching out…I am at my worst.

Complicating the scenario further, the mentor’s video froze and began glitching because her internet connection became unstable. Kaya spent approximately 5 minutes attempting to cope with the problem alone, when eventually the workshop moderator entered his breakout room. However, because she could only view what Kaya elected to screenshare via Zoom–Blender–she could not determine the problem. Kaya stands up from his seat and paces in front of the webcamera. The moderator looks at Kaya’s surroundings and asks Kaya if he is at his mother or father’s house and he responds he is at his mother’s house. Kaya gestures to someone off camera and the viewer sees it is his mother, who has arrived to join him approximately 11 minutes after the start of the mentor’s video freezing, and the two of them decide it is best for him to exit the online session, which he does. The mentor and the moderator debrief about what transpired and the mentor discloses, “That’s when I wish I could be there in person with him.”

### Achieving intersubjectivity

Matthew is a male in his early twenties. The quality of his speech, including the pitch of his voice, speech prosody, and sentence structure can make it difficult at times to understand the words he says. Indeed, the automated Zoom transcript that was generated for his sessions often missed his statements completely, because it could not discern that someone had said something. For his workshop, he is using Procreate.

Matthew’s first session begins with him logged in via his webcam-enabled laptop, but he experiences a typical technical problem for Zoom meetings because of the default settings [44]: his camera is turned off. The mentor asks him if his video is turned on, to which he replies, ‘yes.’ However, his video is not visible. Matthew’s father arrives and resolves the technical issue, but new ones arise. The mentor begins to introduce herself and set the stage for the following weeks, but Matthew’s statements appear to be misaligned with hers:

Mentor: Nice to meet you Matthew…we’re gonna be making our posters for the next few weeks on Procreate.

Matthew: I’m on the basement computer.

Mentor: You’re on the basement computer. And then are you gonna use Procreate on your? Do you have an iPad that you’re gonna use it on?

Matthew: I have an iPad and then I’m going to use it.

While Zoom affords the ability to leverage the visual channel of communication, it is limited by what can be shown on the screen. In this scenario with Matthew, intersubjectivity, which is a shared understanding between individuals [45], is hampered in part by missing visual cues, specifically the mentor being unable to see that Matthew has an iPad because it is out of view from his laptop’s webcam. Moreover, Matthew’s sentence structure further hinders intersubjectivity, even with his father, who is physically present and attempting to assist Matthew in setting up for this first session:

Matthew’s father: Can you share [the iPad] with [mentor] or do you need to work on your laptop?

Matthew: We can share.

Matthew’s Father: OK. You know more than I do.

Mentor: There’s always some sort of technical difficulties, right?

Matthew: OK, then join me on a small zoom.

Mentor: What did you say?

Matthew’s father: I’m not sure what [Matthew’s] trying to do. He’s trying to rejoin the meeting on his iPad, I don’t know.

Matthew joins the Zoom meeting via the iPad while remaining logged into the meeting via the laptop, which is the intended setup for Procreate workshop sessions. He was able to solve a subset of the technical difficulties on his own. However, his sentence structure coupled with imperfect visual channels during videoconferencing appears to have kept others unaware of his intentions.

Other times, Matthew’s use of the affordances of Zoom appear to facilitate achieving intersubjectivity. Toward the end of a session, Matthew began initiating conversation with his mentor and asked, “Do you remember Bing and Bong’s *Tiny Planets*?” At first, the mentor had difficulty apprehending what he said and responded, “Say that one more time. What was that, Matthew?” Matthew repeated his utterance and the mentor replied, “I do not, I feel like I’ve seen it before, but it’s deep in my memory.” At this point, Matthew screenshares an artwork he completed previously and said, “it mysteriously disappeared in 2003.” The mentor stares at the artwork Matthew screenshared and asks, “Was it, I’m guessing, was it a TV show?” Matthew responds affirmatively and clarifies it was a children’s show. Matthew’s speech quality made it difficult for the mentor to understand his reference. Further inhibiting intersubjectivity was that the reference was to a twenty-year-old show that was not very popular (it ran 65 episodes over a span of a year and a half). However, the affordance of screensharing allowed the mentor to infer that the reference was to a television show, thereby facilitating intersubjectivity.

### Sharing during screensharing

Albert is a male in his mid-twenties using Photoshop for his workshop. Albert engages in behavior that suggests he has low self-esteem [46], whereby he repeatedly says Sir’ when referring to the mentor, who is of a similar age to him, and apologizes by saying ‘Sorry’. For example, in the first workshop, Albert starts to search for images in the Bing search engine and the mentor says, “I don’t know Bing very well as a search engine. I more so know Google” and then Albert pivots to Google and says, “And just, sorry.”

Because Albert appears to have low self-esteem, this can hinder his rate of self-disclosure, which tends to be low among those with low self-esteem [47], and thereby also hinder his rapport building with the mentor [48, 49]. The default user interface of Zoom appears to further preclude such rapport building via disclosure because it draws attention to the design project rather than to the other meeting participants present. Specifically, while screensharing, the default user interface displays the video of the shared screen–most frequently the design project in these workshops–at a dimension that is much larger than the displayed videos of the participants’ cameras. When multiple videos are displaying simultaneously on a screen, attention may be drawn more to the larger dimension of the shared screen, especially if it occupies a significant portion of the screen or if it is positioned in a more prominent location [50, 51]. As such, Zoom’s user interface design typically draws focus to the more visually prominent screenshare video rather than either of the participant’s videos, thereby steering conversation toward the project. This user interface also discourages visual understanding of the other participants’ surroundings which may be a source of unintentional disclosure, for example, disclosure through the items the participant has in their Zoom background [52].

The heightened focus on Albert’s project can serve to prevent intimacy building disclosures. In the second workshop, the mentor expresses willingness to work on designing the project to align with Albert’s interests in dinosaurs by saying, “We can do whatever you want. If you’re feeling the [dinosaur] scales, we can definitely do that.” However, Albert conveys reluctance and says, “I… just don’t wanna, like, go off topic from class.” Prominent on Albert’s screen was his design project that entailed adding fur to characters, which may explain why he felt incorporating scales and effectively sharing his interests in dinosaurs with the mentor was “off topic.”

Later in the third workshop the Zoom user interface seems to facilitate riskier disclosures by Albert. He asked his mentor if he could show the inspiration for his design project and turns to YouTube while screensharing. Albert already had a YouTube tab open on another video. After hovering on the tab several times, seemingly hesitating, Albert clicks on it showing a video of the soundtrack from the *My Little Pony* movie and says, “Yeah, I’m a *My Little Pony* fan.” The mentor replies, “Yeah, there’s nothing wrong with that. I know plenty of people who like *My Little Pony*.” Because *My Little Pony* is a show catered to preteen girls, Albert risks ridicule by disclosing his status as a fan. The mentor, however, states he knows ‘plenty of people’ who are fans, thereby normalizing Albert’s interest and implying that the mentor would not ridicule him. Zoom’s user interface may have facilitated this riskier disclosure. By rendering the participants’ videos smaller than the computer screen being shared, fear of ridicule due to disclosures may be lessened. This interpretation accords with previous studies showing engaging in online communication encourages individuals to disclose deeper personal details and discuss sensitive topics that ultimately enrich the depth of their friendships [49]. Moreover, other research shows online communication provides a platform for individuals with low self-esteem to establish and nurture relationships in a secure environment where they feel comfortable expressing their authentic selves [49]. Indeed, after this disclosure event, Albert displays dramatically decreased usage of the words ‘Sir’ and ‘Sorry’ implying that Albert’s level of intimacy with the mentor has increased. For example, in the fifth workshop, Albert once again acts in a manner implying low self-esteem, apologizing for not completing as many things as he wanted to complete:

Albert: I don’t think we’ll be able to get this done because there’s still a lot more things to do, and it’s my fault there. I think there are times I’ve been lazy already being deliberately distracted, skipping, doing this.

Mentor: No, you’re not. You’re not lazy at all. You’ve gotten a ton done.

Albert: I guess maybe I should say distracted.

Mentor: Then yeah, it’s OK. I mean, sometimes you have days where you’re just more productive than others and. That’s totally fine. One thing that I try to remind myself of too, is to remember to be kind to yourself.

Albert: Thanks. Thanks, [Mentor’s name].

Here, however, Albert does not use ‘Sir’ or ‘Sorry’ as was typical for him and instead uses the mentor’s name as he thanks him for his accepting response.

## Discussion

To enhance extant knowledge about experiences with online learning among neurodiverse youth, we presented a qualitative analysis of video recordings from a longitudinal study of neurodiverse youth learning digital art design on the Zoom platform. The rich details observed from the video recordings enabled developing insights into how the same neurodiverse youth can have both positive and negative experiences with online learning. The findings highlight the need for research into online learning to design analyses that unravel what is at times counteracting forces, because otherwise incorrect conclusions may be drawn about online learning [e.g., 53]. From this analysis of an underutilized data source (video recordings of the same student over time), we offer suggestions for designing and implementing online learning opportunities for neurodiverse youth.

A key takeaway includes leveraging technological affordances to facilitate coping with frustrations but also ensuring additional supports are in place when technologies themselves become the source of frustration. Being fluent in the technologies being used certainly helps reduce the likelihood of frustration, but there were times when mentors like Kaya’s did not know or recall how to achieve a desired effect in the design software. In the absence of perfect proficiency, leveraging affordances can be used to curtail escalating frustration. The specific solutions for Kaya and his mentor were the mentor multitasking by searching for the solution online on her own device while Kaya remained focused on the design software screen and using the ‘undo’ option to correct their mistakes. When technology does fail, to limit the spiraling of the situation as Kaya experienced, it seems important for those delivering the lessons (the mentor and moderator in the case of these digital art workshops) to know: 1) where the student is located; and 2) who to contact when the student requires in person support. Neither the moderator nor mentor knew whether Kaya was at his mother or father’s house, which appeared to aggravate his situation because it unnecessarily extended the time it took for someone to reach him in person.

We identified evidence showing how a video conferencing platform like Zoom can both aggravate and mitigate disruptions to the communication flow that may surface because of a neurodiverse student’s reliance on a communication aid (e.g., the Rapid Prompting Method). Video conferencing platforms like Zoom provide the affordance of synchronous communication, but we saw that relying on a communication aid introduced delays in communication by and to Gerardo. However, the unintentional sharing of one’s personal home–like the pet cat–is likely to disrupt the flow of communication regardless of whether a meeting attendee relies on a communication aid. This produced opportunities for Gerardo to ‘catch up’ to everyone else. Besides synchronicity, there exists other ways beyond just technological affordances for people communication online to feel as if they are together and sharing an experience [54]. This includes the perception of similar backgrounds, like demographics, and mutually focusing on others, such as a “Zoom bomb” by a pet, child, or other household member [40]. A key takeaway, then, is to embrace unintended disruptions, so that it is not only the use of a communication aid that is hindering synchronous communication. While sharing one’s personal space via video can raise privacy concerns [55], there may be other ways to leverage affordances of the videoconferencing software to align communication. For example, encouraging the use of a chat feature and stopping on occasion to read the typed comments may signal that asynchronous communication is normative even in a videoconference environment. This may in turn assuage any concerns among those who rely on communication aids regarding disrupting the conversation flow.

The excerpts also showed how online learning can further reduce intersubjectivity (shared understanding) beyond just what is lacking from a neurodiverse youth’s verbal statements, but also ways to enhance it. While video is richer in its ability to convey cues than other online learning modalities that rely solely on voice or text [56], Matthew’s experiences show how this potential can get diminished when a student’s verbal statements are brief. Aggravating the situation further was that neither the mentor nor guardian present who was assisting Matthew with his technology setup appeared certain that he was using Procreate. Here, a suggested solution is to confirm in advance with caregivers regarding the requisite technology setup, but this of course risks adding to the caregiver burden [11, 57]. An alternative solution comes from Matthew’s actions later. We saw with Matthew how screensharing can help achieve intersubjectivity, as when he made a reference to a television show with which the mentor was unfamiliar. Returning to the example of the lack of shared understanding while Matthew was setting up his technology, this suggests asking the student to share what he is attempting to do, such as by holding up the iPad to the camera so that the mentor could confirm what the student is attempting and offer advice.

Our analysis revealed design considerations for the videoconferencing platform used during online learning. During screensharing, the default user interface for Zoom tends to steer focus onto the screen being shared, because that video is of a larger dimension than the video for the meeting participants. We saw with Albert that this may both hinder and facilitate disclosure, depending on the situation. Having the option to select the size of the screens may better cater to neurodiverse students’ needs for more or less intimacy with other meeting attendees, thereby matching their preferences to the situation. Zoom’s side-by-side mode, which can be accessed in the Zoom settings under ‘Share Screen’, allows users to freely manipulate the size of the screen share with the participants’ video feeds simultaneously playing side by side [58]. Further control over the position of videos, such as with YouTube’s ‘Picture in Picture’ video functionality may be useful [59]. Additionally, providing built-in tools or prompts to encourage self-regulation and self-awareness throughout a meeting could help students navigate the complexities of online interactions more effectively, particularly because their needs may change throughout a meeting. These tools may include reminders to take breaks from the current project and prompts to adjust screen settings for optimal engagement. By implementing these design considerations and providing accompanying training resources, videoconferencing platforms can create more supportive and accommodating environments for neurodiverse learners, while being attentive to the fact that online learning is not a static experience.

## Limitations

Readers should interpret findings in light of the study’s limitations. Recordings of online art design workshops were conducted during a period in which Creative Oasis was also offering in-person workshops. As such, data collected represent those who opted into the online modality. While this risks steering the experiences away from being negative toward positive, it is notable that we nonetheless observed a range of negative experiences. Another limitation is that the sample of neurodiverse youth is homogenous with respect to ethnoracial and gender identity. While the homogeneity is reflective of the population served by Creative Oasis, this limits generalization and may even miss detrimental experiences with online learning [60]. For example, one study of Chinese children with ADHD found girls were more likely than boys to experience difficulties with online learning [61]. Other limitations to generalizability is that we do not have access to participants’ neurodiverse condition and participants self-selected into the workshop. Lastly, because the subject was digital art design, some of the experiences observed may not be readily transferable to other subjects.

## Conclusion

Despite the limitations, the study contributes novel insights into the myriad factors shaping experiences with online learning among neurodiverse youth. Through a longitudinal qualitative design, we advance the extant scholarship by demonstrating how the same neurodiverse youth can have both positive and negative experiences with online learning. A key takeaway from the idiographic approach is that online learning is not a monolithic experience, but rather can change dynamically based on the situation encountered. Our approach underscores the need for researchers to use methods to unpack myriad factors, as these may at times operate in opposing ways and thereby mask the overall online learning experience. This may include longitudinal observational methods like the current study, as well as analyses of quantitative data to model potential opposing factors distinctly [53]. Regarding the implications for practitioners, we echo others who underscored the importance of individualized support, flexible learning environments, and strong relationships with educators and caregivers in promoting positive outcomes for neurodiverse individuals [19, 20]. We offer additional suggestions regarding the design and implementation of online learning for neurodiverse youth, which include considerations of both the social (e.g., finding mutual points of focus beyond the task at hand) and technical (e.g., normalizing asynchronous forms of communication, such as the chat function, and using screen sharing features to enable shared understanding) environments that envelop the learning experience. The next steps for future research include sharing findings with organizations that serve neurodiverse communities, comparing the effectiveness of our suggestions to existing practices and designs (e.g., the default screensharing settings for popular videoconferencing platforms like Zoom) on learning experiences both qualitatively and quantitatively, and working with larger samples of neurodiverse youth to identify potentially moderators such as neurodiverse group, gender, and race.
